# The impact of COVID-19 pandemic on access to medical services and its consequences on emergency surgery

**DOI:** 10.3389/fsurg.2023.1059517

**Published:** 2023-04-26

**Authors:** Giovanni Pirozzolo, Bao Riccardo Quoc, Chiara Vignotto, Livio Baiano, Alfredo Piangerelli, Claudia Peluso, Rubina Palumbo, Fabrizio Giuseppe Maria Cimino, Guido Meneghetti, Alberto Grassetto, Maurizio Rizzo, Gabriele Giuseppe Maria Viola, Francesco Fiumara, Marco Scarpa, Alfonso Giovanni Recordare

**Affiliations:** ^1^ General and Emergency Surgery Unit, Dell’Angelo Hospital, AULSS3 Serenissima, Venice, Italy; ^2^ Clinica Chirurgica I, Department of Surgical, Oncological, and Gastroenterological Sciences, University of Padova, Padova, Italy; ^3^ Department of Surgical, Oncological, and Gastroenterological Sciences, University of Padova, Padova, Italy; ^4^ Anesthesiology Department, Dell’Angelo Hospital, AULSS3 Serenissima, Venice, Italy; ^5^ Tbilisi State Medical University (TSMU), Tbilisi, Georgia

**Keywords:** emergency surgery, peritonitis, surgical outcome, complications, survival, COVID-19, pandemic

## Abstract

**Background:**

On March 9, 2020, the Italian Prime Minister announced the lockdown, which was officially closed on May 4. This extraordinary measure was necessary to contain the COVID-19 pandemic spread in Italy. During this phase, a significant decrease in patients' access to Emergency Department (ED) was observed. Delayed access to treatment determined a delay in the diagnosis of acute surgical conditions, as already documented in other clinical areas, with consequences on surgical outcome and survival. Aim of this study is to provide a detailed description of abdominal urgent-emergent conditions surgically treated and surgical outcomes during the lockdown in a tertiary referral Italian hospital, compared with historical data.

**Methods:**

A retrospective review of urgent-emergent patients surgically treated in our department was conducted in order to compare patients' characteristics and surgical outcomes during the period March 9th—May 4th, 2020 with the same period of the previous year.

**Results:**

152 patients were included in our study, 79 patients in 2020 group and 77 patients in 2019. We found no significant differences between the groups regarding ASA score, age, gender, and disease prevalence. Significant differences were found in symptom duration before ER access and abdominal pain as the main symptom in non-traumatic conditions. We also performed a sub-analysis on peritonitis which showed significant differences in: hospital length of stay, presence of colostomy vs. ileostomy, and fatal events in 2020. No differences were found in the use of laparoscopy.

**Conclusions:**

While the overall number of ER accesses has decreased in 2020 group, the number of patients surgically treated in emergency-urgency conditions has not decreased. However, those patients waited significantly more before the hospital access. This diagnostic delay was associated with a more severe clinical condition and a consequent significantly worse prognosis.

## Introduction

In 2020, on March the 9th, the Italian Prime Minister announced the lockdown, which was officially closed on May the 4th. This extraordinary measure was necessary to contain the COVID-19 pandemic spread in Italy (1, 2). Healthcare systems were deeply reorganized, in order to both hold the burden of the pandemic and guarantee essential health services, with direct consequences on the organization of surgical activity (3, 4).

During this phase, patients' access to Emergency Department (ED) dramatically decreased, as already documented during the Taiwan SARS Epidemic in 2003 (5–8). This population's behavior, strongly motivated by the common perception of Hospitals as hotspots for infection, could have led to severe consequences. For example, the Italian Pediatric Hospital Research Network reported a small series of 12 cases of delayed access in severe conditions resulting from fear of COVID-19 infection (9), and similarly, a dramatic increase in the rate of out-of-hospital cardiac arrest was reported (10). Moreover, data from ISTAT (Statistical Italian Institute) revealed an increased overall mortality in the period between 20 February and 31 March 2020, if compared with the average of the previous years (11).

As well as cardiologic emergencies, emergency surgery outcomes could have been significantly affected by diagnostic delay due to the lockdown (12–14). In fact, a survey from the ACOI network showed a significant reduction in urgent interventions and an unusual delay in the presentation of non-traumatic abdominal emergencies (15). Moreover, a retrospective study about surgical emergencies documented a 32% reduction in the number of people presenting at the ED, compared with the previous year (16). A WSES survey documented the increased incidence of severe septic abdominal abscesses during the pandemic, especially for appendicitis and cholecystitis (16). However, clear data about the consequences of delayed access in ED to emergency surgery during the Pandemic are not available.

The aim of this study is to provide a detailed description of surgical emergencies and outcomes during the lockdown in a tertiary referral Italian hospital, compared with historical data from the same center.

## Materials and methods

### Study design

This is an observational case-control study on the effect of COVID19 pandemic on emergency surgery delay. This study was carried out in accordance with the principles of Helsinki, and the study was notified to the Ethical Committee of the AULSS3 Serenissima. The Case group was composed by a consecutive series of acute patients undergoing surgery for urgent and emergent conditions in the General Surgery Unit of the Dell'Angelo Hospital, Venice, during the lockdown imposed by the Italian government due to the COVID-19 outbreak, from March 9th to May 4th. We retrospectively collected data regarding age, sex, previous comorbidity, ASA score, symptoms at hospital admission and their duration, laboratory findings, diagnosis, type and duration of surgery, Intensive Care Unit (ICU) length of stay, overall length of stay, presence, and type of stoma, need of reoperation and survival. We also collected data about the pre-hospital delay, defined as the time between the appearance of symptoms and the admission to the emergency department. The same data were also collected from the same period of the previous year (Control Group), in order to compare the two groups of patients.

### Inclusion criteria and outcome measures

All patients who underwent urgent/emergency surgical procedures during the study period were included, without age limitations. Intraperitoneal phlogistic diseases (i.e., diverticulitis, appendicitis, cholecystitis) were included in the peritonitis subgroup. Bowel obstruction, bowel ischemia and trauma patient were included in separate subgroups. All the patients who underwent elective surgery, such as surgical oncology procedures, or patients whose surgical details were not completely available were excluded.

The primary outcome was patient's 30-day survival and mortality. The secondary outcomes were the presence of stoma, rate of re-operation, ICU length of stay, and overall length of in-hospital stay.

### Admission and management protocols during COVID19 pandemic

During COVID-19 Pandemic, patients were referred to our surgical Unit after SARS-CoV2 molecular testing and standard chest x-ray. In the early phase of the Pandemic, a Chest CT scan was routinely performed. The severity of the urgency was evaluated case by case, and patients with severe, not postponable disease, were operated before the molecular testing results. After the surgical operation, they were located and monitored in an isolated area until the molecular testing results. SARS-CoV-2 RNA testing was routinely performed, on all patients, every 2 or 4 days during the hospitalization. In case of symptoms such as fever and cough the test was anticipated. All healthcare personnel was screened every week.

### Surgical techniques

During the early phase of the COVID-19 pandemic, we followed the recommendation of surgical societies on the management of emergency and urgent patients during COVID-19 pandemic (17). In particular, the special recommendations regarding the use of laparoscopy (18) were followed: use a closed suction system; use of balloon trocars to avoid smoke leakage; avoid the evacuation of fumes; suction of the entire pneumoperitoneum at the end of the procedure before removing the trocars or before conversion to open surgery; in case of a lack of skills and adapted materials, laparoscopy would better be avoided, especially in an emergency setting.

Considering all these recommendations, appendectomy was performed laparoscopically in all cases. Cholecystectomy was performed, if patients presented within the golden 72 h, mostly with a minimally invasive approach.

### Statistical analysis

Continuous data are presented as median (IQR) and categorical data are presented as *n* (%). Continuous variables are compared with Student's *t*-test, Mann-Whitney test, or Kruskal-Wallis test as appropriate. Categorical variables are compared in 2 × 2 contingency tables using Chi-square test and Fisher exact test. *P* values less than 0.05 were considered statistically significant. Comparison between the two groups was performed using SPSS Statistics, version 26.

## Results

### Patients' characteristics

Overall, 152 patients were included in our study, 79 patients in 2020 case group and 77 patients in the 2019 control group. The median age was 53 years in 2020 and 60 years in the control group, male female ratio was 45/34 and 47/30 respectively. Patients' characteristics are summarized in Table 1a.

**Table 1:** a: patients’ characteristics, b: admission symptoms, c: laboratory results, d: diagnosis.

	2020 (79 pts)		2019 (77 pts)		
**a. Patients Characteristics**					
	Median	IQR	Median	IQR	*p*
Age	53	50	60	43	
	n/n	%	n/n	%	
M/F	45/34	M57%	30/47	M61%	
	*n*	%	*n*	%	*p*
ASA 1	33	41,8	29	37,7	0,600
ASA 2	22	27,8	29	37,7	0,191
ASA 3	19	24,1	16	20,8	0,624
ASA 4	3	3,8	3	3,9	0,974
ASA 5	2	2,5	0	0	0,164
anticoagulants	4	5,1	8	10,4	0,289
antiplatelets	12	15,2	10	13,3	0,558
**b. Symptoms**					
	*n*	%	*n*	%	*p*
Prehospital delay (days)	1,50	5,00	1,00	0,00	0,056
Abdominal pain	79	100,00	70	90,86	0,006
Fever	23	29,10	21	27,25	0,860
Asthenia	0	0,00	3	3,89	0,118
Jaundice	1	1,26	0	0,00	0,322
Dysphagia	0	0,00	1	1,29	0,310
Nausea/vomiting	37	46,62	26	20,76	0,096
Stipsis	17	21,42	11	9,08	0,239
GI bleeding	7	10,08	5	6,49	0,579
Diarrhoea	1	1,26	1	1,30	0,985
**c. laboratory results**					
	*n*	%	*n*	%	*p*
PCR (admission)	5,80	26,93	2,47	19,90	0,648
WBC (admission)	10,85	7,85	10,87	8,18	0,389
PCT (admission)	0,21	18,15	0,28	3,83	0,214
**d. Diagnosis**					
	*n*	%	*n*	%	*p*
Peritonitis	45	57,0	35	45,5	0,151
Bowel obstruction	24	30,4	19	24,7	0,425
Proctologic emergencies	6	7,6	13	16,9	0,076
GI bleeding	1	1,3	3	3,9	0,299
Bowel ischemia	1	1,3	3	3,9	0,299
Trauma	0	0,0	4	5,2	0,040
Other	2	1,9	0	0,0	0,160

**Figure 1:**
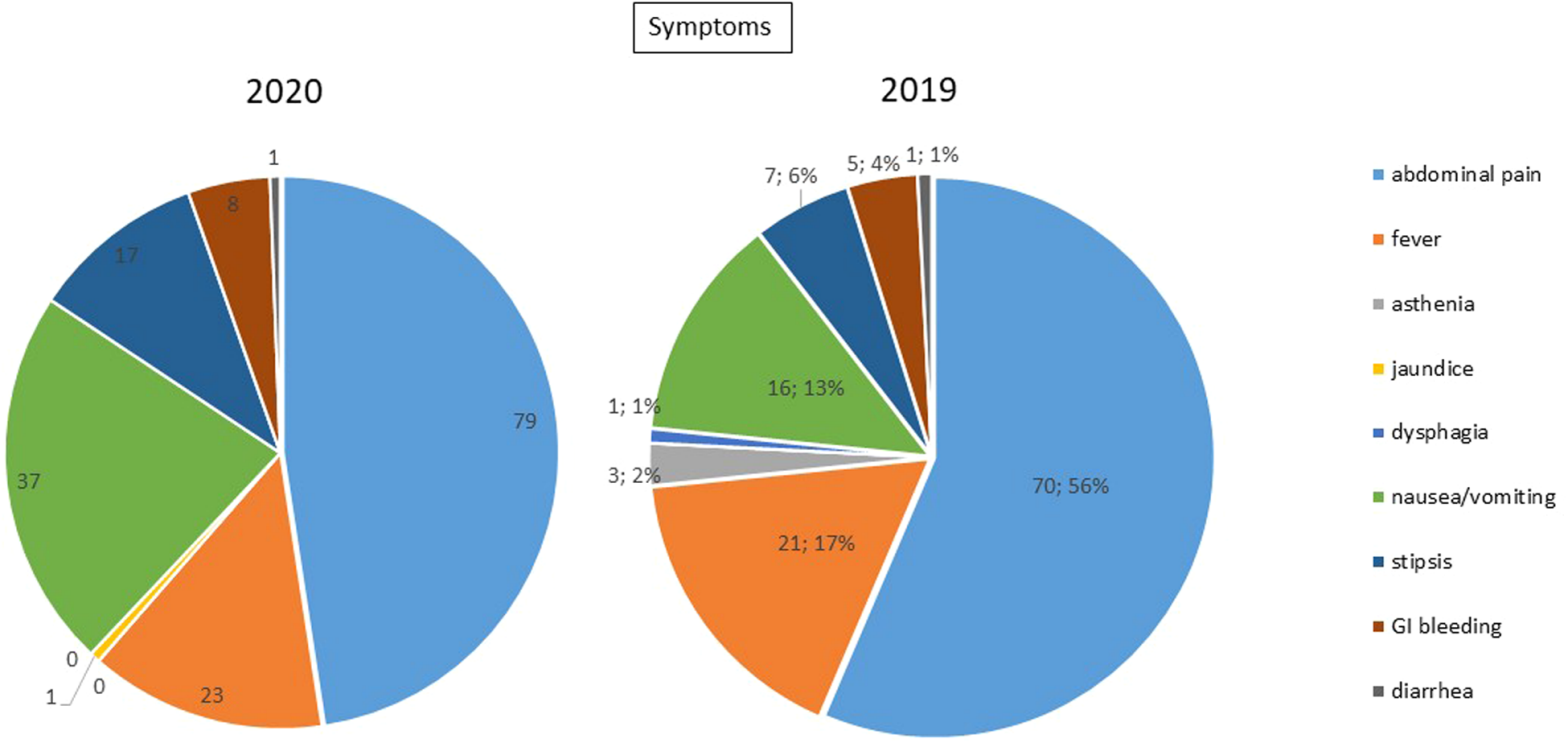
Symptoms.

**Figure 2:**
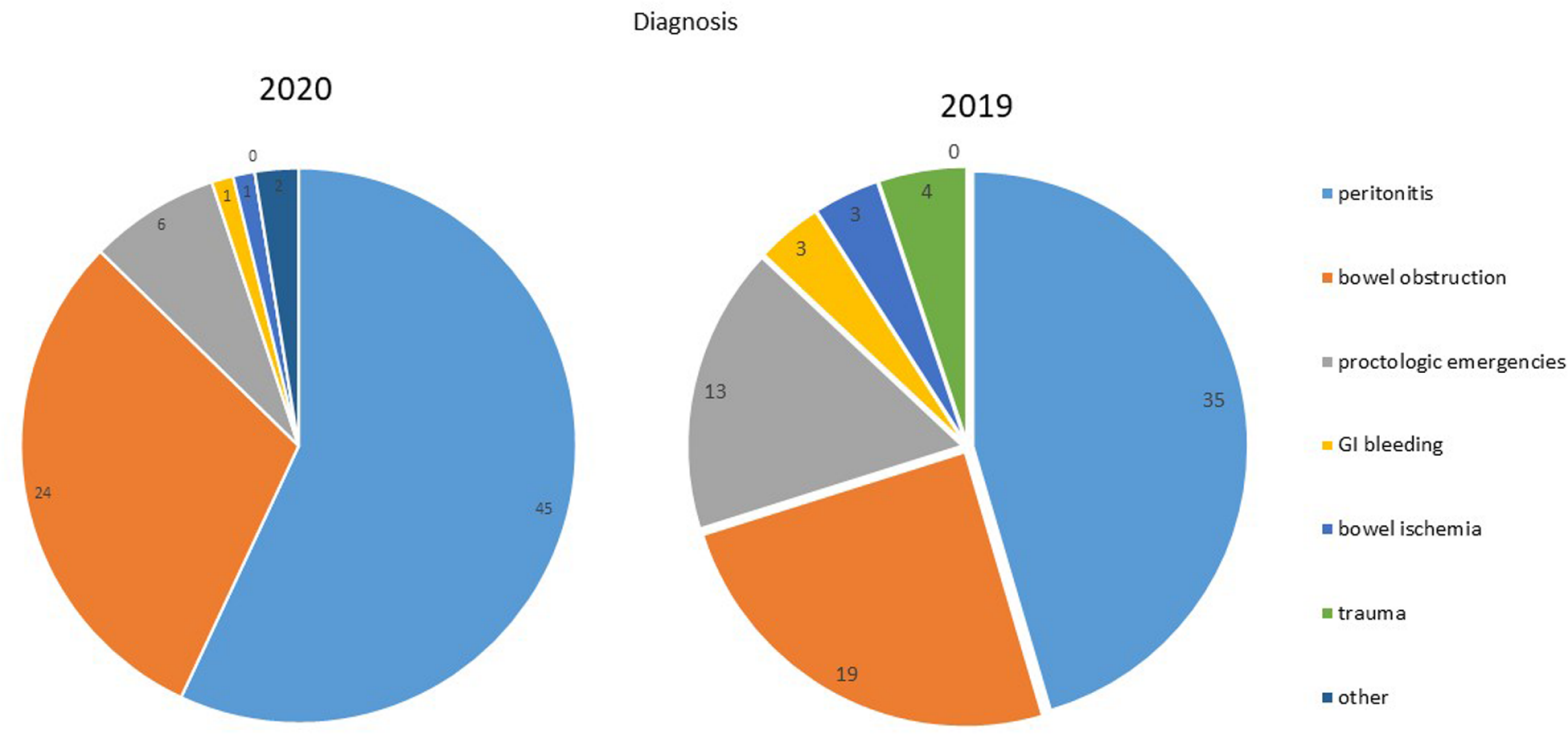
Diagnosis.

**Figure 3:**
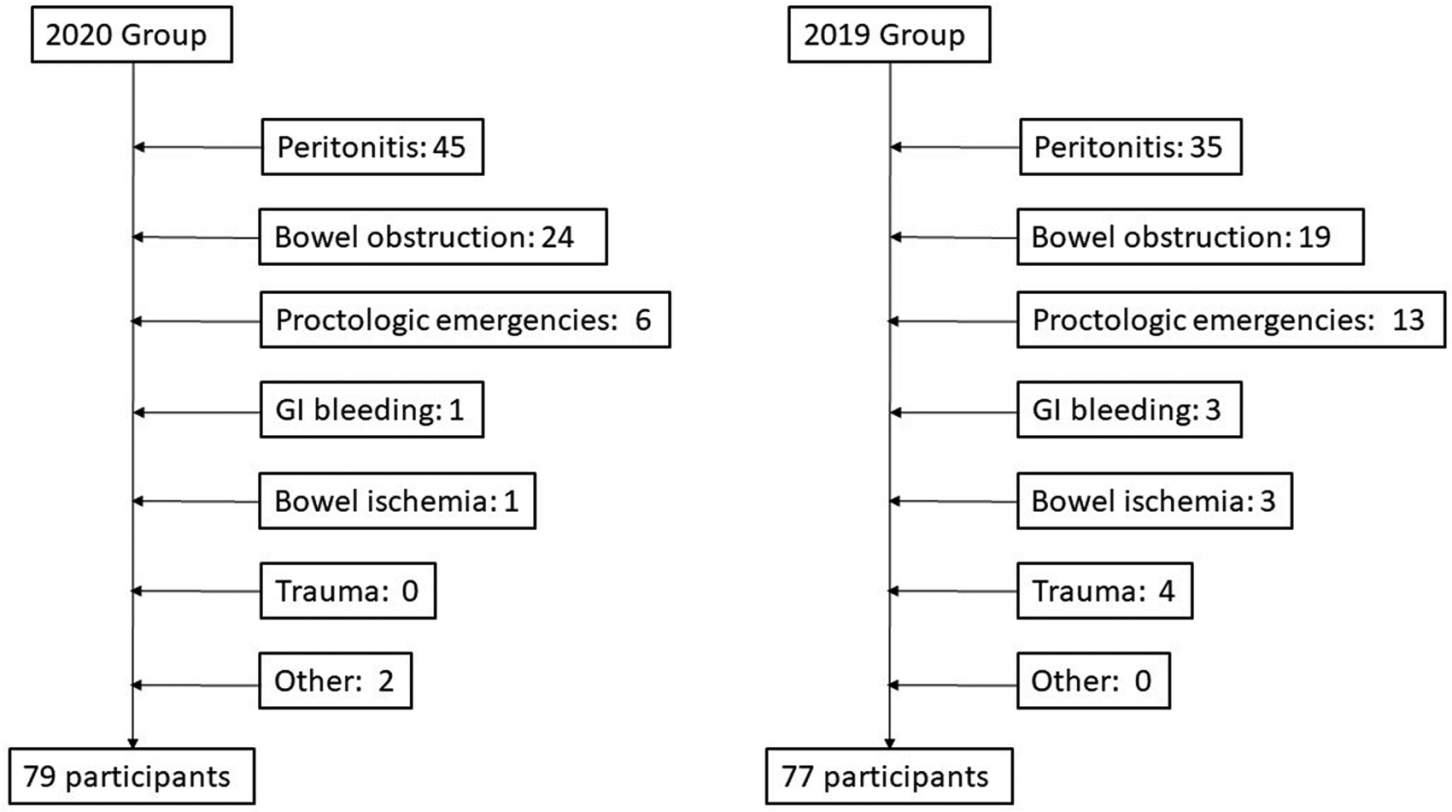
flow chart.

Upon hospital admission, 100% of patients reported abdominal pain in the case group, and 90.9% of patients in the control group; similarly, nausea, vomiting, constipation and fever were more frequent in the case group than the control group (symptoms are summarized in Table 1b, Figure 1). In both groups, peritonitis was the most common condition, even if more frequent in the 2020 group. The second more frequent condition was bowel obstruction, followed by proctologists emergencies, trauma, gastrointestinal bleeding, and bowel ischemia (Figures 2, 3). Contingency tables showed significant differences for abdominal pain (79 vs. 70 in 2020 and 2019 respectively, *p* = 0.006) and a statistically significant reduction of trauma patients in 2020 (0 vs. 4, *p* = 0.04). The overall results are summarized in Table 1.

**Figure 4:**
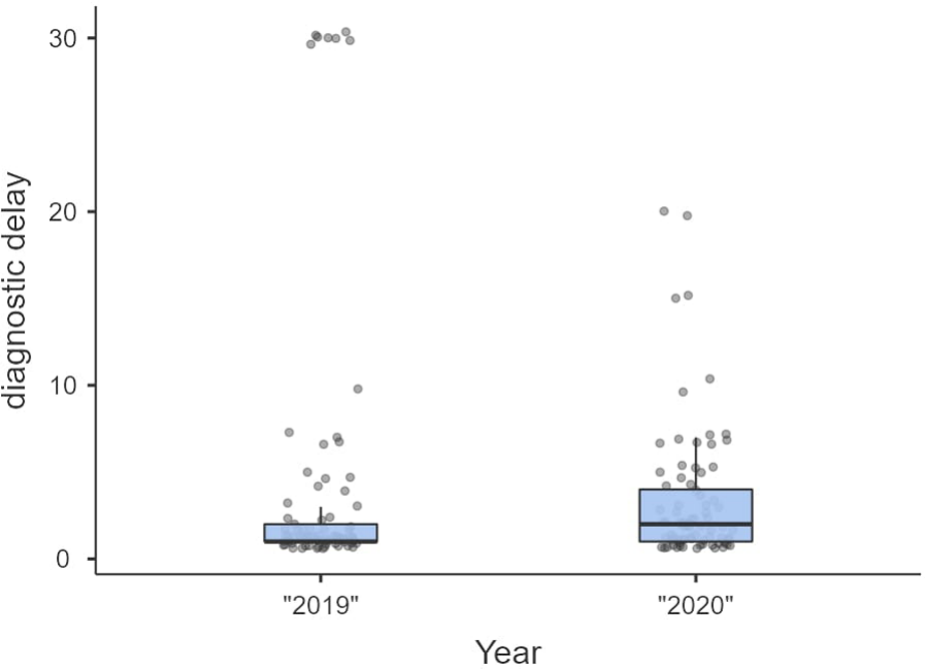
Diagnostic delay.

**Figure 5:**
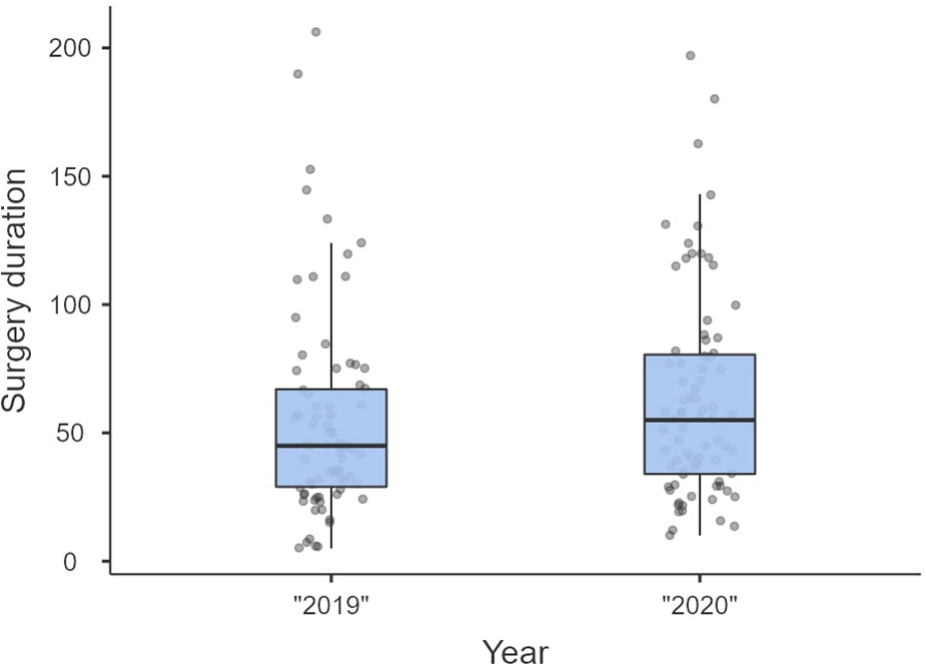
Surgery duration.

**Figure 6:**
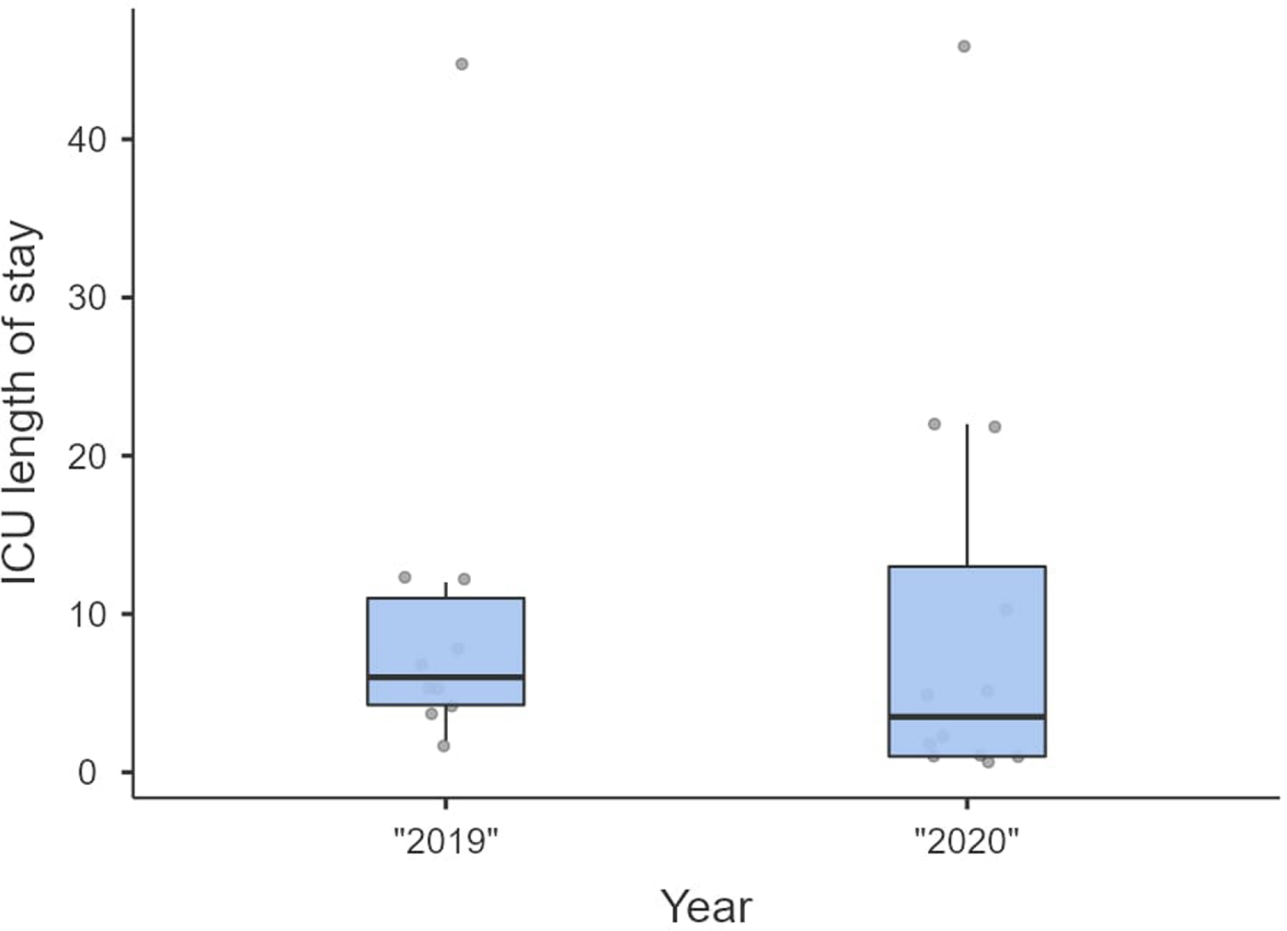
ICU length of stay.

**Figure 7:**
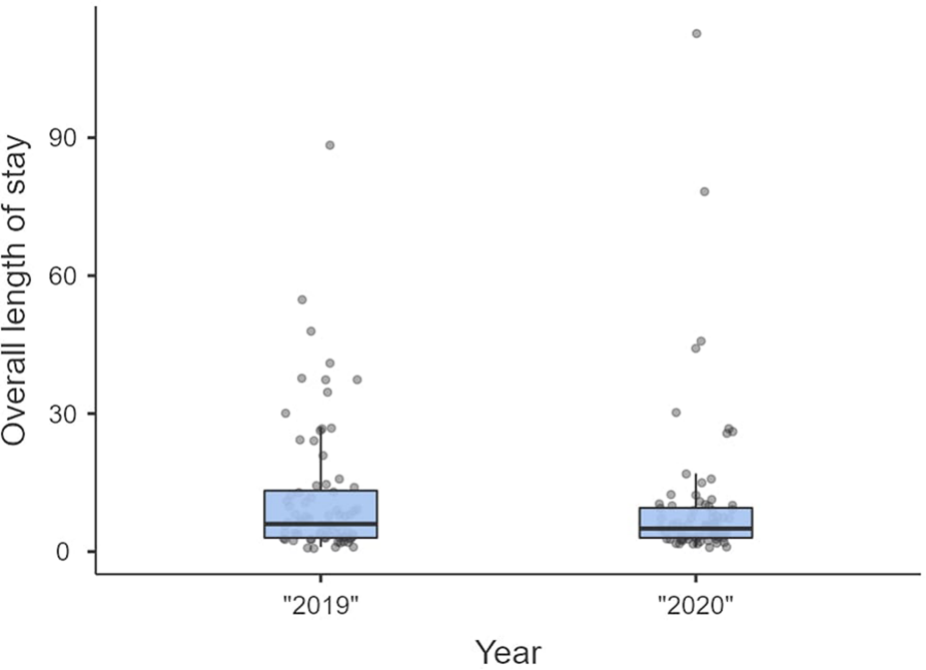
Overall length of stay.

Non-significant differences were shown for diagnostic delay (Figure 4), surgery duration (Figure 5), ICU length of stay (Figure 6), overall length of stay (Figure 7), and mortality. Overall results are summarized in Tables 2, 3.

**Table 2:** Overall outcomes (1).

	2020		2019		*p*
	Median	IQR	Median	IQR	
Prehospital delay (days)	1,5	5	1	0	0,056
Surgery duration (minutes)	71	61	49	60	0,119
ICU length of stay (days)	3,5	15	6	8	0,314
Overall length of stay (days)	15,5	36	15,5	25	0,231

**Table 3:** Overall outcomes (2).

	2020		2019		*p*
	*n*	%	*n*	%	
Re-operation	3	6,7	2	5,7	0,449
Ileostomy	0	0	3	8,6	0,207
Colostomy	2	4,4	0	0	0,045
Deaths	5	6,33	3	3,89	0,491

### Peritonitis subgroup

We conducted a sub-analysis on peritonitis patients, which showed a significant delay before hospital access (median 2 vs. 1 day in 2020 and 2019 respectively, *p* = 0.013) (Table 4), a significantly increased mortality (5 vs. 0 in 2020 and 2019 respectively, *p* = 0.042), but a less frequent ileostomy in 2020, (0 vs. 3 in 2020 and 2019 respectively, *p*: 0.207) with more frequent colostomies (2 vs. 0 *p* = 0.045) (Table 5).

**Table 4:** Peritonitis subgroup outcomes (1).

	2020		2019		*p*
	Median	IQR	Median	IQR	
Prehospital delay (days)	2	4	1	0	0,013
PCR (admission)	14,7	23,3	0,5	14,9	0,686
WBC (admission)	14	8,3	13,3	11,1	0,902
PCT (admission)	0,25	0,98	0,56	5,32	0,164

**Table 5:** Peritonitis subgroup outcomes (2).

	2020		2019		*p*
	*n*	%	*n*	%	
Re-operation	3	6,7	2	5,7	0,449
Ileostomy	0	0	3	8,6	0,207
Colostomy	2	4,4	0	0	0,045
Deaths	5	11	0	0	0,042

No significant differences in laboratory findings, use of minimally invasive surgical techniques, duration of surgery, ICU length of stay, overall length of stay, and re-operation rate between the two groups were found.

### CoVID19 detection

We found only one patient, treated for emergency surgery in that period, positive for the COVID-19 RNA test, completely asymptomatic for SARS-CoV. The patient, treated for appendicitis, was recovered in the COVID ward after the surgical treatment, and he didn't report either surgical postoperative complications or COVID-19-related consequences.

## Discussion

During the 2020 lockdown, even if the overall number of ED accesses decreased, the number of patients surgically treated in emergency-urgency conditions did not decrease in an Italian tertiary referral center for emergency and trauma surgery. The overall analysis showed a statistically significant difference in abdominal pain prevalence as the main symptom and the complete absence of trauma patients in the 2020 group. This last result is easily understandable since this was a direct consequence of the travel limitation imposed by the Italian government. On the other hand, the data on pain as the main symptom finds a more complex explanation, which could be clarified by the sub-analysis results.

Peritonitis' sub-analysis showed that, in our series, 2020 patients waited significantly more before hospital access, as already hypothesized in other studies (7, 16) compared to 2019. A similar situation had already been described during SARS Epidemic in Taiwan in 2003 (6). Thus, this behavior is, very likely, a direct consequence of the fact that hospitals were perceived, especially during the early phase of the pandemic, as unsafe places, despite emergency cares never stopped on the national territory. Indeed, health facilities and, consequently, health workers were the most affected by the COVID-19 infection, as reported by Italian Istituto Superiore di Sanità (ISS) and by the Chinese experience (19).

The effects of delayed diagnosis in emergency conditions, especially in abdominal sepsis, are well known, as well as morbidity and mortality are time-dependent factors in those patients (20, 21). This diagnostic delay was, consequently, associated with a more severe clinical condition and a significantly worse prognosis. The most evident consequence was the dramatic increase in postoperative mortality in peritonitis patients in the 2020 group. Moreover, the lower incidence of ileostomy and the increased frequency of Hartmann's procedures performed in 2020 could be a direct consequence of more severe peritonitis found in this group of patients. On the other hand, secondary to a safer approach favored by surgeons to avoid unnecessary complications and re-interventions in a phase of great stress for the hospital, especially the intensive care units. Therefore, the cost of the pandemic was not only paid in terms of direct deaths from SARS-CoV2 infection, but also paid in terms of deaths due to delayed emergency condition treatments.

Nevertheless, the surgical approach did not change the use of minimally invasive techniques, despite the recommendations of some surgical societies (18), without consequences on the intra-hospital spread of COVID-19. Indeed, none of the staff of our Department, nor the patients, tested positive for the COVID-19 swab test during the lockdown. Actually, only one patient treated for emergency surgery, was resulted positive for the admission swab test, but it was completely asymptomatic. He was isolated in a COVID area before and immediately after surgery, and he has never passed through the General Surgery ward that was kept safe.

The retrospective design, the heterogeneity, the complexity of conditions and treatments, and the relatively small sample are the main limits of the study. However, this sample size allowed us to show the differences in terms of diagnostic delay and mortality. Moreover, even if the conclusions of the study are limited to the Italian Health System, a similar behavior was observed during the Taiwan SARS epidemic, in completely different conditions (5–8). We should consider that, in the early phase, all the involved Countries were equally unprepared and the national scientific societies produced similar guidelines. Furthermore, the modality of transmission and the consequences of the disease were not fully known and an effective vaccine had not yet been developed, which resulted in extremely cautious behavior of patients and institutions.

In conclusion, our results seem to confirm some previous observations about the severe consequences of diagnostic delay, in emergency surgery, during the early phase of the pandemic outbreak. Those findings could help health authorities to consider adequate countermeasures in order to guarantee hospital access to urgent-emergent non-pandemic conditions, in a difficult situation such as a pandemic outbreak.

## Data Availability

The raw data supporting the conclusions of this article will be made available by the authors, without undue reservation.
